# Supplementary material for: TGFβ1 Suppressed Matrix Mineralization of Osteoblasts Differentiation by Regulating SMURF1–C/EBPβ–DKK1 Axis
Source: Int J Mol Sci. 2020 Dec 21;21(24):9771. doi: 10.3390/ijms21249771 (PMC7767413; doi:10.3390/ijms21249771)
Supplement: Supplementary file 1 [file ijms-21-09771-s001.pdf]

Supplementary Figure Legends

TGFβ1 suppressed matrix mineralization of osteoblasts differentiation by regulating SMURF1-C/EBPβ-DKK1 axis.

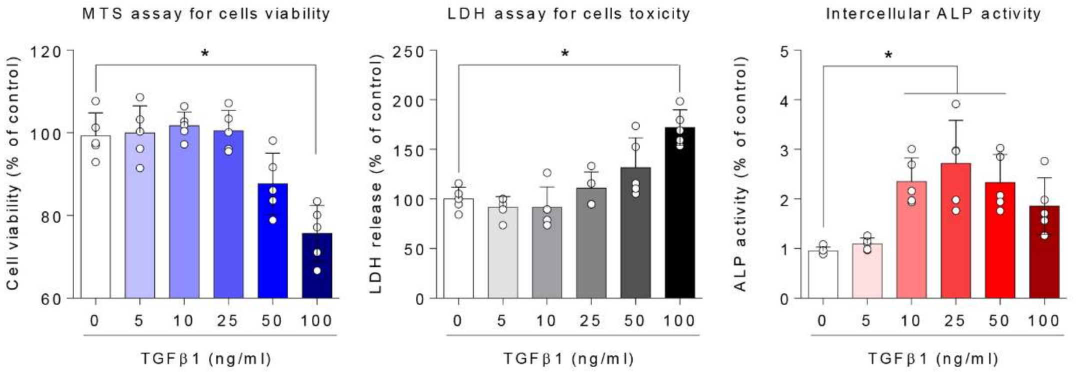

Figure S1. Effect of TGFβ1 on osteoprogenitor cells. Osteoprogenitors were treated with various dose of TGFB1 for 3 days and analyzed by (A) MTS assay, (B) LDH assay, and (C) intercellular ALP activity. Values are expressed as the mean ± SD. **p*<0.05.

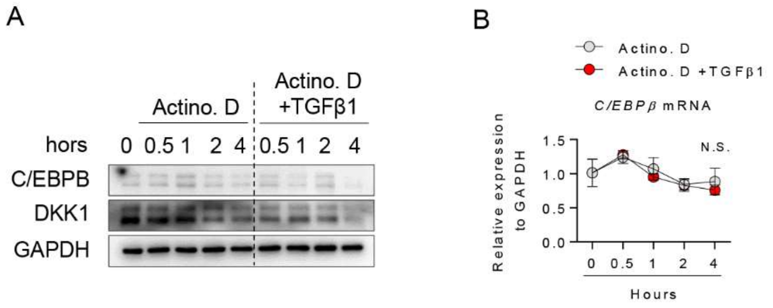

Figure S2. TGFβ1 had no effect on C/EBPβ transcription level in osteoprogenitors. Osteoprogenitors were pretreated with actinomycin D for 30 min, followed by treatment with 10 ng/ml TGFβ1 for indicated times, and analyzed by (A) immunoblotting and (B) qPCR. Immunoblotting images and qPCR data are representative from the three independent experiments. N.S., Not significant.

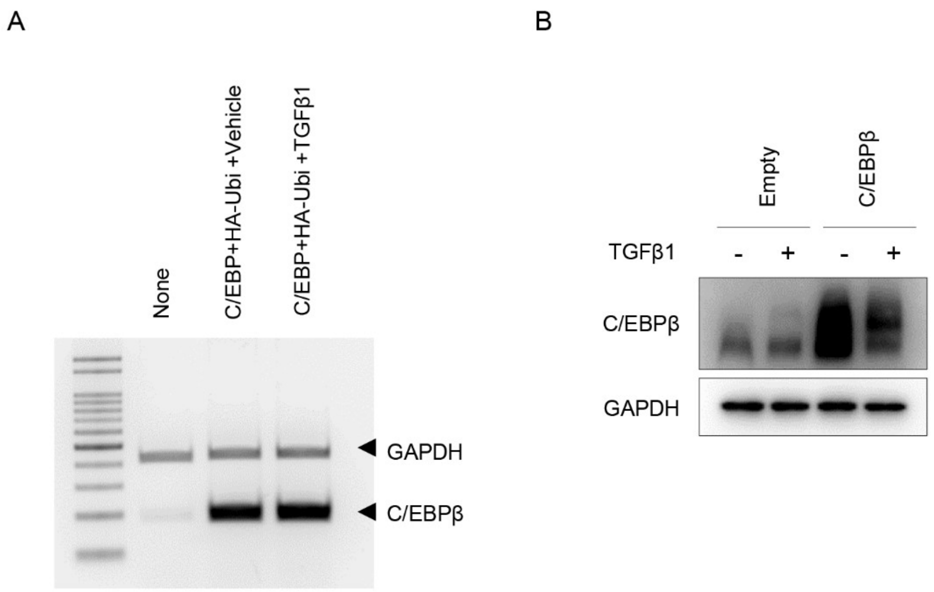

Figure S3. TGFβ1 decreased C/EBPβ protein expression. 293T cells were co-transfected with C/EBPB and HA-Ubi plasmids for 24 h, followed by treatment with 10 ng/ml TGFβ1 for 24 h, and analyzed by (A) RT-PCR and (B and C) immunoblotting. RT-PCR and immunoblotting images are representative from the three independent experiments.

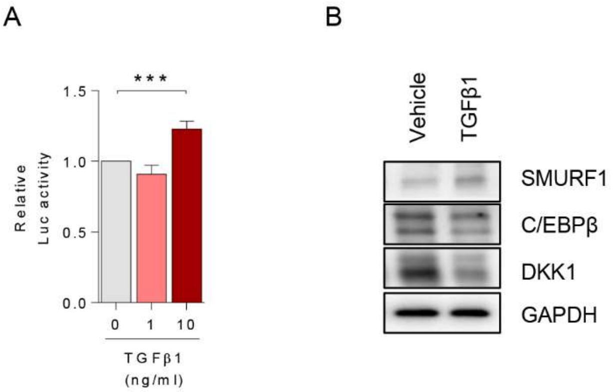

Figure S4. TGFβ1 induced SMURF1 promoter. MG63 cells were transfected with SMURF1 promoter and analyzed by (A) luciferase assay. (B) MG63 cells were treated with 10 ng/ml TGFβ1 for 24 h, and analyzed by immunoblotting. Luciferase data are representative from the five independent experiments.

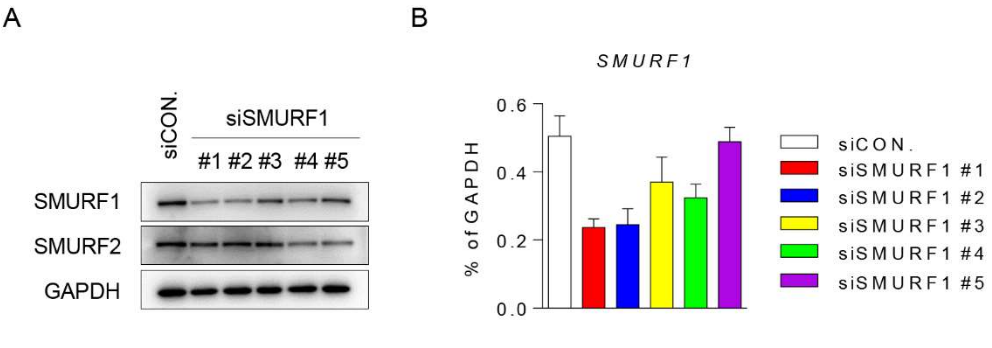

Figure S5. Suppression efficiency of siSMURF1 in osteoprogenitors. Osteoprogenitors were transfected with sicontrol (siCON) or 5 types of siSMURF1, analyzed by (A) immunoblotting and (B) qPCR. Immunoblotting images and qPCR data are representative from the three independent experiments.

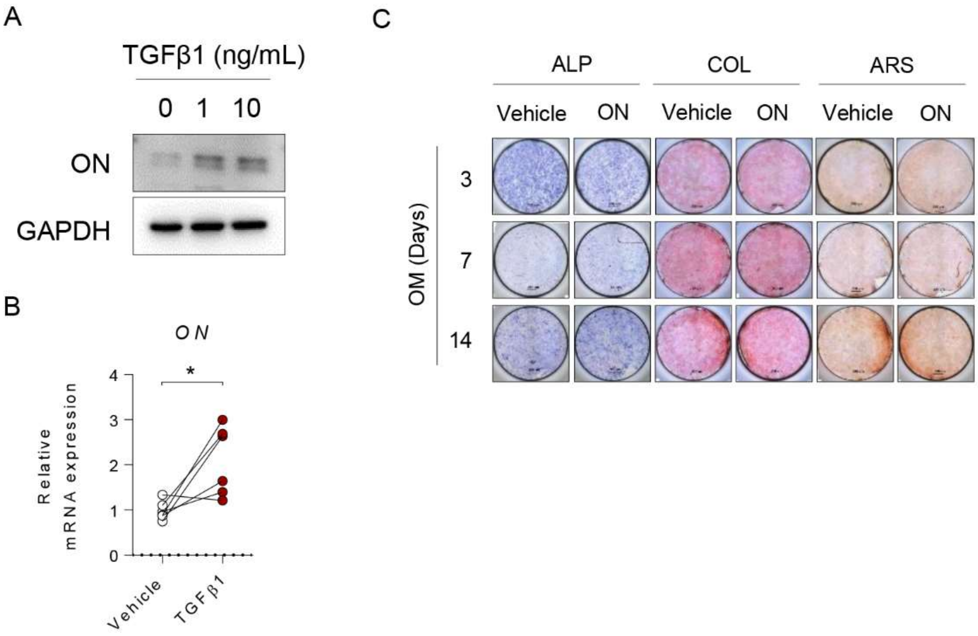

Figure 6. Osteonectin did not affect osteoblastic differentiation. Osteoprogenitors were treated with TGFβ1 for a day as indicated and analyzed by (A) Immunoblotting and (B) qRT-PCR. The stimulated osteoprogenitors were stimulated with vehicle or 10 ng/ml osteonectin during osteoblast differentiation. As indicated days, we performed various staining. Scale bar is 200 μm. All data are representative from the six independent experiments. Values are expressed as the mean ± SD. *p<0.05.
